# The differentiation of mesenchymal stem cells to vascular cells regulated by the HMGB1/RAGE axis: its application in cell therapy for transplant arteriosclerosis

**DOI:** 10.1186/s13287-018-0827-z

**Published:** 2018-04-03

**Authors:** Xiaohu Meng, Min Chen, Wenjie Su, Xuan Tao, Mingyang Sun, Xiaoping Zou, Rongchao Ying, Wei Wei, Baolin Wang

**Affiliations:** 1 Department of General Surgery, The Second Affiliated Hospital of Nanjing Medical University, No.121 Jiangjiayuan, Nanjing, 210011 China; 2 Department of Gastroenterology, Nanjing University Medical School Affiliated Nanjing Drum Tower Hospital, Nanjing, China; 3 Department of Gastroenterological Surgery, Hangzhou First People’s Hospital Affiliated to Nanjing Medical University, Hangzhou, China

## Abstract

**Background:**

Mesenchymal stem cell (MSC) transplantation shows promise for treating transplant arteriosclerosis, at least partly via promoting endothelial regeneration. However, the efficacy and safety are still under investigation especially regarding recent findings that neointimal smooth muscle cells are derived from MSC-like cells. The high mobility group box 1 (HMGB1)/receptor for advanced glycation end-product (RAGE) axis is involved in regulating proliferation, migration, and differentiation of MSCs, and therefore it can be presumably applied to improve the outcome of cell therapy. The aim of the current study was to investigate this hypothesis.

**Methods:**

Rat MSCs were treated with HMGB1 or modified with HMGB1 vectors to activate the HMGB1/RAGE axis. RAGE was targeted and inhibited by specific short hairpin RNA vectors. We assessed the capacity for cell proliferation, migration, and differentiation after vector transfection in vitro and in a rat model of transplant arteriosclerosis. The expression of CD31 and α-smooth muscle actin (αSMA) was determined to evaluate the differentiation of MSCs to endothelial cells and smooth muscle cells.

**Results:**

Exogenous HMGB1 treatment and transfection with HMGB1 vectors promoted MSC migration and vascular endothelial growth factor (VEGF)-induced differentiation to CD31^+^ cells while inhibiting their proliferation and platelet-derived growth factor (PDGF)-induced differentiation to αSMA^+^ cells. Such an effect was blocked by RAGE knockdown. HMGB1-modified cells preferably migrated to graft neointima and differentiated to CD31^+^ cells along with significant relief of transplant arteriosclerosis and inhibition of HMGB1 and RAGE expression in graft vessels. RAGE knockdown inhibited cell migration to graft vessels.

**Conclusions:**

HMGB1 stimulated MSCs to migrate and differentiate to endothelial cells via RAGE signaling, which we translated to successful application in cell therapy for transplant arteriosclerosis.

**Electronic supplementary material:**

The online version of this article (10.1186/s13287-018-0827-z) contains supplementary material, which is available to authorized users.

## Background

Despite the development of surgical techniques and new immune suppressive agents, chronic allograft rejection remains an obstacle to long-term allograft survival [1]. Transplant arteriosclerosis (TA) as a specific form of arteriosclerosis is typically evident in chronically rejected organs. The affected arteries show a thickening of the intimal layers that are filled with vascular smooth muscle cells (SMCs) and extracellular matrix. The process named as intimal hyperplasia or neointimal formation gives rise to arterial stenosis which restricts the blood supply to grafts with a consequent late graft loss. Therefore, it makes sense to explore effective interventions for TA.

Transplantation of mesenchymal stem cells (MSCs) was introduced to prolong allograft survival with satisfactory outcomes in preclinical and clinical studies [2–7]. The therapeutic effects were initially linked to the immunomodulatory properties of MSCs, including induction of regulatory T cells, secretion of anti-inflammatory cytokines, and suppression of alloantigen reactive lymphocytes. Further research was undertaken to generate durable chimerism and induce immune tolerance by MSC-based therapy [8, 9], although this turned out to be difficult. Other studies revealed that MSC transplantation was effective in treating arteriosclerosis. Neointimal formation was attenuated by MSC transplantation in balloon-induced arterial injury models, which was associated with enhanced endothelial repair [10, 11]. Moreover, transplantation of endothelial-like cells derived from MSCs preferably suppressed intimal hyperplasia following vascular injury [12]. This suggested that MSCs attenuated arteriosclerosis at least partly via endothelial regeneration. However, the safety of MSC-based therapy was queried in recent studies on the origin of neointimal SMCs. Traditionally, it was believed that the key process of neointimal formation included the proliferation and migration of medial SMCs which switched from the contractile to the proliferative or synthetic phenotype in response to vascular injury. But it has now been revealed that multipotent stem cells which reside in vascular walls migrate to the intimal layers of injured vessels and subsequently differentiate into neointimal SMCs [13, 14]. Although the stem cells exist physiologically as a small population, they are capable of self-renewal and proliferation, and some of them acquire an MSC-like phenotype. Whether resident vascular stem cells contribute more than medial SMCs to intimal hyperplasia is still in dispute, however, and further investigation is still ongoing. Nevertheless, this discovery raised concerns about the participation of transplanted MSCs in arteriosclerosis presumably via SMC differentiation.

In this scenario, we have been exploring ways of improving the efficacy of MSC transplantation with a special focus on the regulation of cell differentiation that would promote endothelial regeneration. The current study aimed to evaluate whether the differentiation of MSCs to vascular cells would be influenced by high mobility group box 1 (HMGB1) and whether it could be used to provide favorable outcomes of MSC-based therapy for transplant arteriosclerosis. HMGB1 is an evolutionarily abundant and highly conserved DNA binding protein, and is passively released from necrotic cells or actively secreted by inflammatory cells to the extracellular milieu upon cell stress to regulate innate and adaptive immunity. HMGB1 stimulates the immune response, at least in part, through interaction with its principal binding partner, receptor for advanced glycation end-product (RAGE). Both HMGB1 and RAGE are highly expressed in atherosclerotic plaques to accelerate the progression of atherosclerosis [15]. Moreover, the HMGB1/RAGE axis is instrumental in promoting acute and chronic rejection [16–23]. Despite its proinflammatory properties, HMGB1 promotes tissue repair by recruiting stem cells. For instance, HMGB1 promoted the proliferation, migration, and osteogenic differentiation of MSCs via STAT3 and Ras/MAPK signaling pathways [24, 25]. Cell migration was also enhanced by HMGB1-induced synthesis of the CXCL12 receptor CXCR4, which possessed potent chemotactic activity for MSCs [26]. However, whether HMGB1 regulates the differentiation of MSCs to vascular cells was not investigated, and data on the proliferation and migration of MSCs induced by HMGB1 were not consistent. Two recent studies revealed that extracellular HMGB1 released from necrotic cells and activated platelets inhibited MSC migration to apoptotic cardiac myocytes mediated by hepatocyte growth factor and its receptor MET [27, 28]. Another study demonstrated that RAGE knockout rescued streptozotocin-induced suppression of MSC proliferation and differentiation [29]. Overall, previous studies seem to obtain mutually exclusive results at least partly due to different experimental conditions. Nevertheless, they suggest a diversity and complexity of MSC response to HMGB1 stimulation. Therefore, it is necessary to investigate the role of the HMGB1/RAGE axis on MSC transplantation for TA.

## Methods

### Cell culture and reagents

F344 rat bone marrow MSCs were purchased from Cyagen Biosciences Company (Shanghai, China) and cultured in Dulbecco’s modified Eagle’s medium (DMEM) supplemented with 10% fetal bovine serum (FBS; Gibco, ThermoFisher Scientific). The cells were grown as an adherent culture at 37 °C in a humidified atmosphere of 5% CO_2_ and 95% air. Vascular endothelial growth factor (VEGF) and platelet-derived growth factor (PDGF)-BB were obtained from ProSpec (St. Louis, MO, USA). RIPA lysis buffer, BCA protein assay kit, and CCK-8 kit were purchased from Beyotime Biotech (Shanghai, China). Antibodies together with their manufacturers are listed below: rabbit anti-HMGB1 IgG, rabbit anti-RAGE IgG, mouse anti-CD31 IgG, mouse anti-α-smooth muscle antigen (αSMA) IgG, mouse anti-CD68 IgG, phycoerythrin (PE)-conjugated and fluorescein isothiocyanate (FITC)-conjugated anti-αSMA antibodies, secondary antibodies of Alexa Fluor 488 conjugated goat anti-mouse IgG, Alexa Fluor 488 conjugated goat anti-rabbit IgG, Alexa Fluor 647 conjugated goat anti-mouse IgG and Alexa Fluor 647 conjugated goat anti-rabbit IgG, horseradish peroxidase (HRP) conjugated goat anti-rabbit IgG and HRP-conjugated goat anti-mouse IgG (all from Abcam, Shanghai, China), PE-conjugated and FITC-conjugated anti-CD31 antibodies (Miltenyi Biosciences, Shanghai, China), mouse anti-rat tubulin IgG (BD, Shanghai, China), and mouse anti-FLAG IgG (Cell Signaling Technology, Shanghai, China). Recombinant HMGB1 protein and other reagents were purchased from Sigma-Aldrich unless otherwise stated.

### Lentiviral transfection

All lentiviral vectors were constructed by Genechem (Shanghai, China): HMGB1 expression vector pLV-HMGB1 and its negative control pLV-control, which contained the reporter gene green fluorescent protein (GFP), a set of three RAGE short hairpin RNA (shRNA) vectors including shRNA-RAGE1, shRNA-RAGE2, and shRNA-RAGE3, plus one negative control pshRNA-control which contained the sequence of cherry red fluorescent protein (RFP) (Additional file 1: Supplemental Materials and Methods). Before viral transfection, MSCs were plated in six-well plates at 2 × 10^5^ cells per well and cultured for 24 h to allow cell adhesion. The virus was then added at a concentration of 6 × 10^5^ transduction units per well to the culture medium supplemented with 5 μg/ml polybrene to enhance transfection efficiency. After transfection for 12 h, the medium was replaced with fresh medium, and the cells were cultured for 3 days. The transfected cells were selected by 5 μg/ml puromycin and later propagated in culture medium. Transfection efficiency was assessed by calculating the percentage of fluorescent protein-labeled cells in the total population (Additional file 2: Figure S1). QRT-PCR revealed that the transfected cells retained MSC phenotypes of CD29 and CD90, which was similar to untransfected MSCs (Additional file 1: Supplemental Materials and Methods; Additional file 3: Figure S2).

### Animal model, study groups, and stem cell transplantation

Aorta transplantation was performed between Lewis and F344 rats to establish a TA model. The Lewis-to-F344 strain combination was used for allografts; syngeneic controls were made from the F344 to F344 strain. MSC transplantation started on postoperative day 30, being repeated three times at an interval of 15 days. Stem cells were prepared in serum-free medium and injected via the tail vein at a dose of 2 × 10^6^ cells per rat each time. All recipients were sacrificed on the 90th postoperative day, and the grafts were harvested for histology and immunofluorescence analysis. The segment of vascular anastomoses was carefully removed from the graft specimens to avoid the effect of suture material on intimal hyperplasia. Animal groups and treatment protocols are summarized in Table 1. All animals were purchased from Vital River (Beijing, China) and received humane care in compliance with the ‘Principles of Laboratory Animal Care’ formulated by the National Society for Medical Research and the ‘Guide for the Care and Use of Laboratory Animals’ prepared by the Institute of Laboratory Animal Resources and published by the National Institutes of Health (NIH Publication No. 86-23, revised 1996). The experiment was approved by the Committee of Animal Experiment Ethics at Nanjing Medical University.

**Table 1:** Animal groups and treatment protocols

Group	*n*	Donor/recipient	Cell therapy
Isograft	8	F344/F344	No
Allograft	8	Lewis/F344	No
pLV-control	8	Lewis/F344	Infusion of pLV-control-transfected cells
pLV-HMGB1	8	Lewis/F344	Infusion of pLV-HMGB1-transfected cells
pshRNA-control	8	Lewis/F344	Infusion of pshRNA-control-transfected cells
pshRNA-RAGE1	8	Lewis/F344	Infusion of pshRNA-RAGE1-transfected cells
pshRNA-RAGE3	8	Lewis/F344	Infusion of pshRNA-RAGE3-transfected cells

### Proliferation assay

Cell proliferation was measured by a CCK-8 kit. Firstly, the cells were seeded in 96-well plates at 4 × 10^3^ cells per well. After incubation for 24 h, the cells were cultured in the medium containing 10% CCK-8 solution for 2 h. Finally, the optical density (OD) was detected at 450 nm. The experiments were performed in triplicate for each study group to calculate the average OD value. The cell survival in the treatment groups was represented by the OD value normalized to that of untreated MSCs.

### Transwell migration assay

Cell migration was measured by transwell assay. Firstly, the chambers of 24-well plates were separated into upper and lower compartments by transwell inserts with 8-μm pore membrane filters (Corning, Shanghai, China). Next, the cells were seeded in the upper compartment at 8 × 10^4^ cells per well and incubated for 24 h. The upper compartment contained serum-free medium, while the medium supplemented with 15% FBS was added to the lower compartment to stimulate cell migration. Finally, the cells migrating to the lower side of the membrane were fixed in methanol, stained with 0.1% crystal violet solution, and visualized by phase-contrast microscopy. The experiments were repeated five times for each group, and data are expressed as the average migrating cell count per ×200 field micrograph.

### In vitro differentiation of MSCs to vascular cells

The MSCs were cultured in DMEM supplemented with 5% FBS and 12.5 ng/ml PDGF-BB for 14 days to induce SMC differentiation. PDGF-BB was replaced by 25 ng/ml VEGF to induce endothelial differentiation. The expression of CD31 and αSMA was detected by flow cytometry to identify the cells that differentiated to endothelial cells (ECs) and SMCs, respectively.

### Histological and immunohistochemical analysis

The cross sections of graft vessels were stained with hematoxylin and eosin to evaluate neointima formation. The neointimal thickness was normalized to the full thickness of vessel walls and expressed as a percentage. Other sections were stained by an immunoperoxidase technique to measure the expression of HMGB1 and RAGE. Briefly, the sections were processed through antigen retrieval and blocking procedures and then consecutively incubated with primary antibody, HRP-conjugated secondary antibody, and the substrate solution containing 3,3-diaminobenzidine. The nuclei were counterstained with Mayer’s hemalum solution.

### Evans blue staining

The aortic endothelial barrier integrity was assessed by the Evans blue dye exclusion test as previously described [30]. Briefly, freshly procured graft vessels were perfused with heparinized phosphate-buffered solution to remove blood cells, subsequently with 0.3% Evans blue dye for 5 min, and finally with phosphate-buffered solution to remove the dye residue. The vessels were opened by longitudinally cutting in half and then fixed between two glass slides. The images were photographed by Nikon Coolpix 4500. Positive staining indicated endothelium dysfunction or denudation. The area was quantified with image pro plus analysis software to calculate the ratio of unstained area (stained without dark blue) to the total intraluminal surface area of the graft vessels.

### Cell and tissue immunofluorescence

The expression of HMGB1, RAGE, CD31, and αSMA in the cultured cells and graft vessels was detected by immunofluorescence. Firstly, frozen sections of graft vessels and cultured cells were fixed with cold acetone and blocked by 10% goat serum. Target protein was probed with specific primary antibodies and then visualized by incubation with corresponding fluorescent protein-labeled secondary antibodies. Finally, the nuclei were counterstained with 4′,6-diamidino-2-phenylindole (DAPI).

### Western blot analysis

Total cellular protein was isolated from fresh tissue or cells with a RIPA lysis buffer containing protease inhibitor. Protein concentrations in the supernatants were measured by BCA protein assay. Protein samples were separated using 12% SDS-PAGE gels, transferred to polyvinylidene difluoride membranes, and blocked with 5% nonfat milk solution. The membranes were incubated with primary antibodies and corresponding HRP-conjugated secondary antibodies. Antibody binding was visualized using the ECL system.

### Flow cytometry

CD31 and αSMA on the cell surface were detected by flow cytometry. Briefly, the cells were transferred to 5-ml flow tubes and incubated with PE- or FITC-conjugated antibodies in the dark for 30 min at 4 °C. The cells were then washed and suspended in phosphate-buffered saline. Fluorescence was quantified using BD FACSCanto II flow cytometer (BD Biosciences, CA, USA). The gates were set based on isotype control antibodies.

### Statistical analysis

Statistical analysis of the data was performed using GraphPad Prism 5.0. Data are expressed as mean ± standard deviation and compared between groups by the Mann-Whitney test. A *P* value < 0.05 indicates a significant difference.

## Results

### HMGB1 inhibited MSC proliferation, but promoted their migration through RAGE activation

**Fig. 1:**
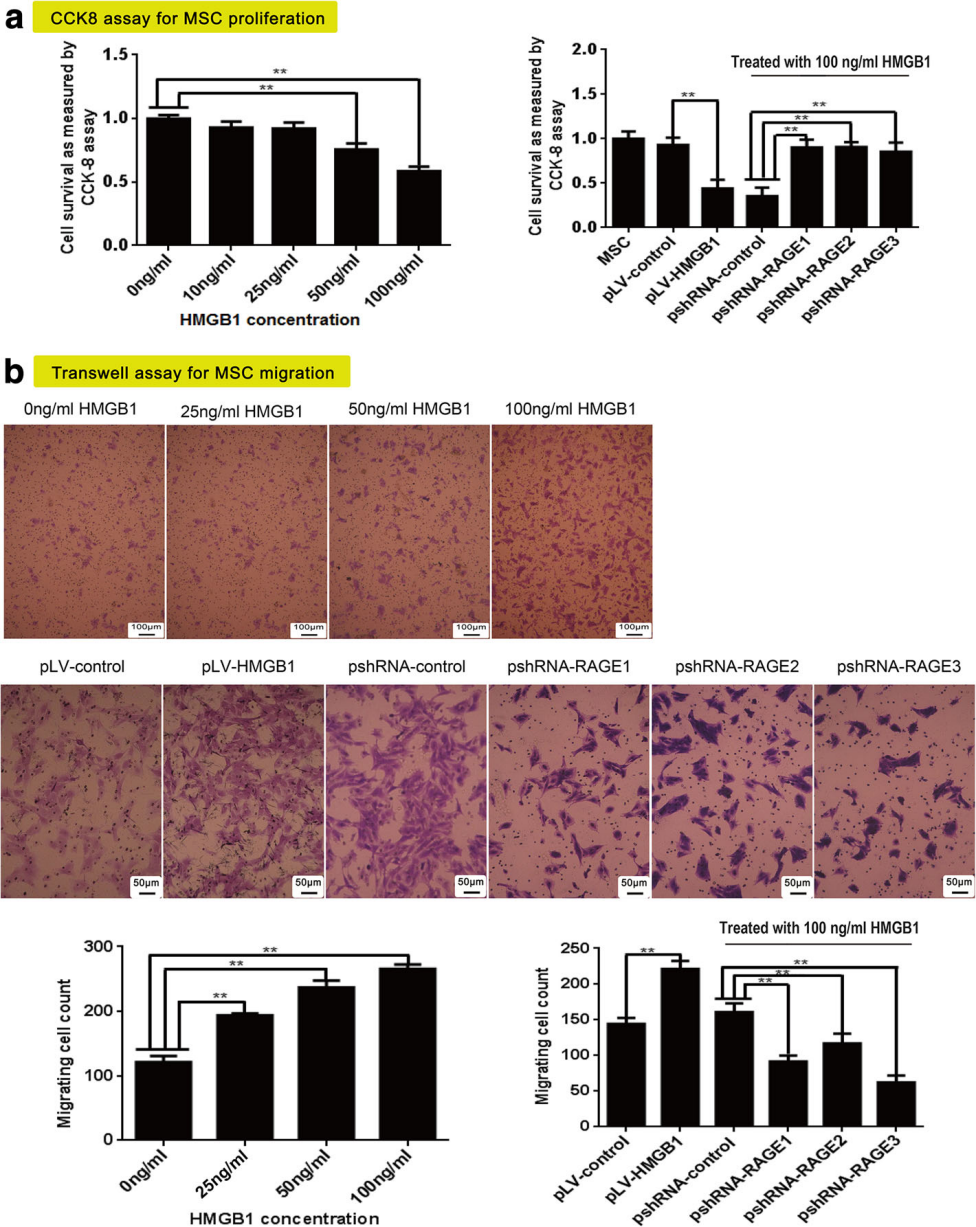
The proliferation and migration of mesenchymal stem cells (MSCs) in response to high mobility group box 1 (HMGB1) stimulation and receptor for advanced glycation end-product (RAGE) inhibition. **a** In CCK8 assay, cell survival was decreased after exogenous HMGB1 treatment and pLV-HMGB1 transfection. However, the cells proliferated with resistance to 100 ng/ml HMGB1 after being transfected with pshRNA-RAGE1, pshRNA-RAGE2, and pshRNA-RAGE3. **b** Transwell assay revealed that cell migration was increased after exogenous HMGB1 treatment and pLV-HMGB1 transfection. However, HMGB1-induced cell migration was inhibited by transfection with pshRNA-RAGE1, pshRNA-RAGE2, and pshRNA-RAGE3. CCK-8 and transwell assay were repeated three and five times, respectively, for each group. Group comparisons were made using the Mann-Whitney test. **P < 0.05

Cell growth was greatly inhibited after HMGB1 was added to the culture medium. Cell survival gradually decreased as the HMGB1 concentration increased at a gradient from 10 ng/ml to 100 ng/ml. Moreover, cell proliferation was inhibited after pLV-HMGB1 transfection. Cell survival was reduced by nearly 50% compared with pLV-control-transfected cells. However, RAGE knockdown attenuated the inhibitory effect of HMGB1. The cells transfected with RAGE shRNAs grew faster than the negative control in culture medium supplemented with 100 ng/ml HMGB1 (Fig. 1). Therefore, HMGB1 inhibited the cell proliferation via a RAGE-dependent pathway. The HMGB1/RAGE axis also regulated MSC migration. The number of migrating MSCs was increased in transwell assay after the cells were treated with recombinant HMGB1 or transfected with pLV-HMGB1. However, RAGE knockdown inhibited HMGB1-induced cell migration. The cells transfected with RAGE shRNAs were less responsive to HMGB1 treatment, and the number of migrating cells was significant lower than the negative control (Fig. 1).

### HMGB1 promoted VEGF-induced differentiation of MSCs to CD31^+^ cells via RAGE activation

**Fig. 2:**
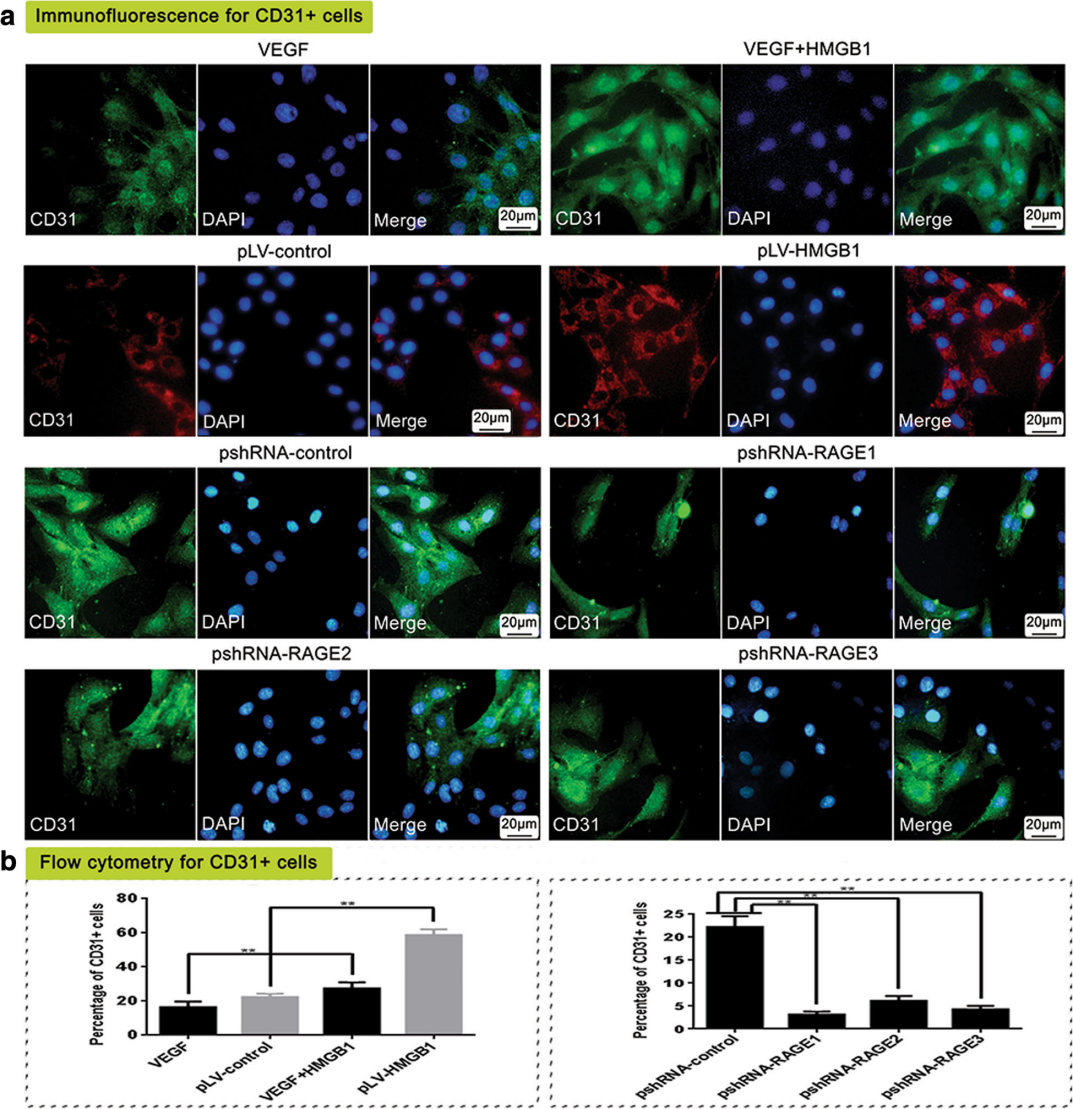
The differentiation of MSCs to CD31^+^ cells in response to high mobility group box 1 (HMGB1) stimulation and receptor for advanced glycation end-product (RAGE) inhibition. The population of CD31^+^ cells was determined by immunofluorescent staining (**a**) and flow cytometry (**b**). Both exogenous HMGB1 treatment and pLV-HMGB1 transfection stimulated vascular endothelial growth factor (VEGF)-induced differentiation of MSCs to CD31^+^ cells. The fraction of CD31^+^ cells increased from 16% to 27% upon 100 ng/ml HMGB1 treatment, and from 21% to 58% after pLV-HMGB1 transfection. However, the stimulatory effect was inhibited by RAGE knockdown. After the cells were transfected with pshRNA-RAGE1, pshRNA-RAGE2, and pshRNA-RAGE3, the percentages of CD31^+^ cells reduced from 22.5% to 3.0%, 6.4%, and 4.0%, respectively. The experiments were repeated three times for each group. Group comparisons were made using the Mann-Whitney test. **P < 0.05. DAPI 4′,6-diamidino-2-phenylindole

After MSCs were treated with 25 ng/ml VEGF for 14 days, 16% of cells differentiated to CD31^+^ cells. The percentage of CD31^+^ cells was significantly increased to 27% upon additional treatment with 100 ng/ml HMGB1. pLV-HMGB1 transfection also promoted endothelial differentiation and increased the population of CD31^+^ cells from 21% to 58%. However, the cells transfected with RAGE pshRNAs acquired resistance to HMGB1 stimulation, and the proportion of CD31^+^ cells was dramatically reduced from 22.5% to approximately 5% (Fig. 2).

### HMGB1 inhibited PDGF-induced differentiation of MSCs to αSMA^+^ cells via RAGE activation

**Fig. 3:**
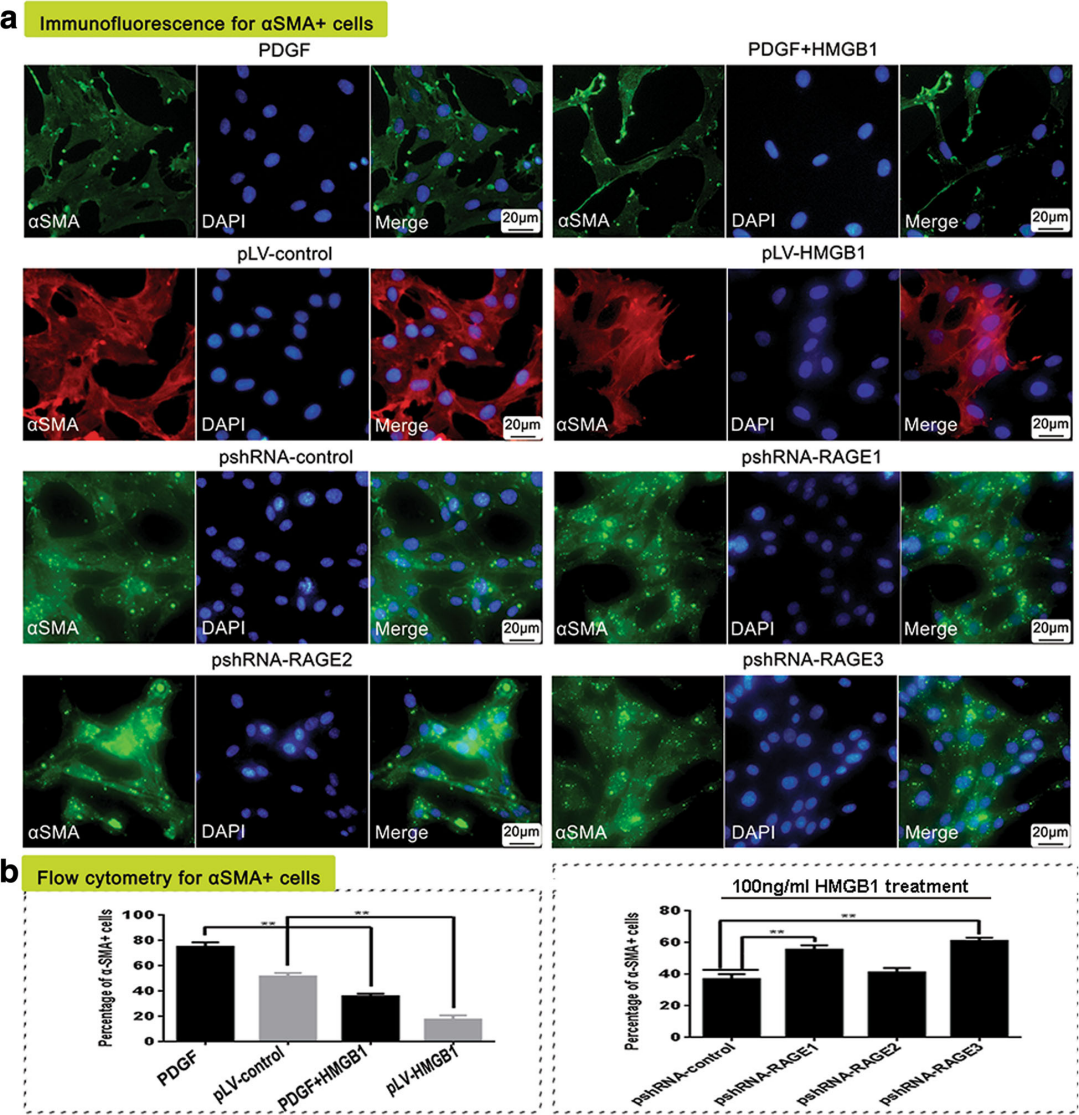
The differentiation of MSCs to α-smooth muscle actin (αSMA)^+^ cells in response to high mobility group box 1 (HMGB1) stimulation and receptor for advanced glycation end-product (RAGE) inhibition. The population of αSMA^+^ cells was determined by immunofluorescent staining (**a**) and flow cytometry (**b**). Both exogenous HMGB1 treatment and pLV-HMGB1 transfection inhibited platelet-derived growth factor (PDGF)-induced differentiation of MSCs to αSMA^+^ cells. The fraction of αSMA^+^ cells was decreased from 75% to 36% upon 100 ng/ml HMGB1 treatment, and from 53% to 18% after pLV-HMGB1 transfection. However, the inhibitory effect was blocked by RAGE knockdown. After the cells were transfected with pshRNA-RAGE1 and pshRNA-RAGE3, the percentages of αSMA^+^ cells increased from 38% to 55% and 61%, respectively. pshRNA-RAGE2 transfection had little effect. The experiments were repeated three times for each group. Group comparisons were made using the Mann-Whitney test. **P < 0.05. DAPI 4′,6-diamidino-2-phenylindole

Seventy-five percent of MSCs differentiated to αSMA^+^ cells after the cells were incubated with the medium containing 12.5 ng/ml PDGF-BB for 14 days. The differentiated cells were remarkably reduced to 36% of the total population upon additional treatment with 100 ng/ml HMGB1. Moreover, pLV-HMGB1 transfection inhibited cell differentiation to αSMA^+^ cells compared with the negative control, and the proportion of αSMA^+^ cells was reduced from 53% to 18%. However, cell transfection with pshRNA-RAGE1 and pshRNA-RAGE3 attenuated HMGB1 inhibition on PDGF-induced cell differentiation. The percentage of αSMA^+^ cells increased from 38% to 55% and 61% (Fig. 3). Thus, HMGB1 inhibited PDGF-induced cell differentiation depending on RAGE activation.

### Transplantation of pLV-HMGB1-transfected MSCs remarkably ameliorated TA along with inhibition of HMGB1 and RAGE expression in allograft vessels

**Fig. 4:**
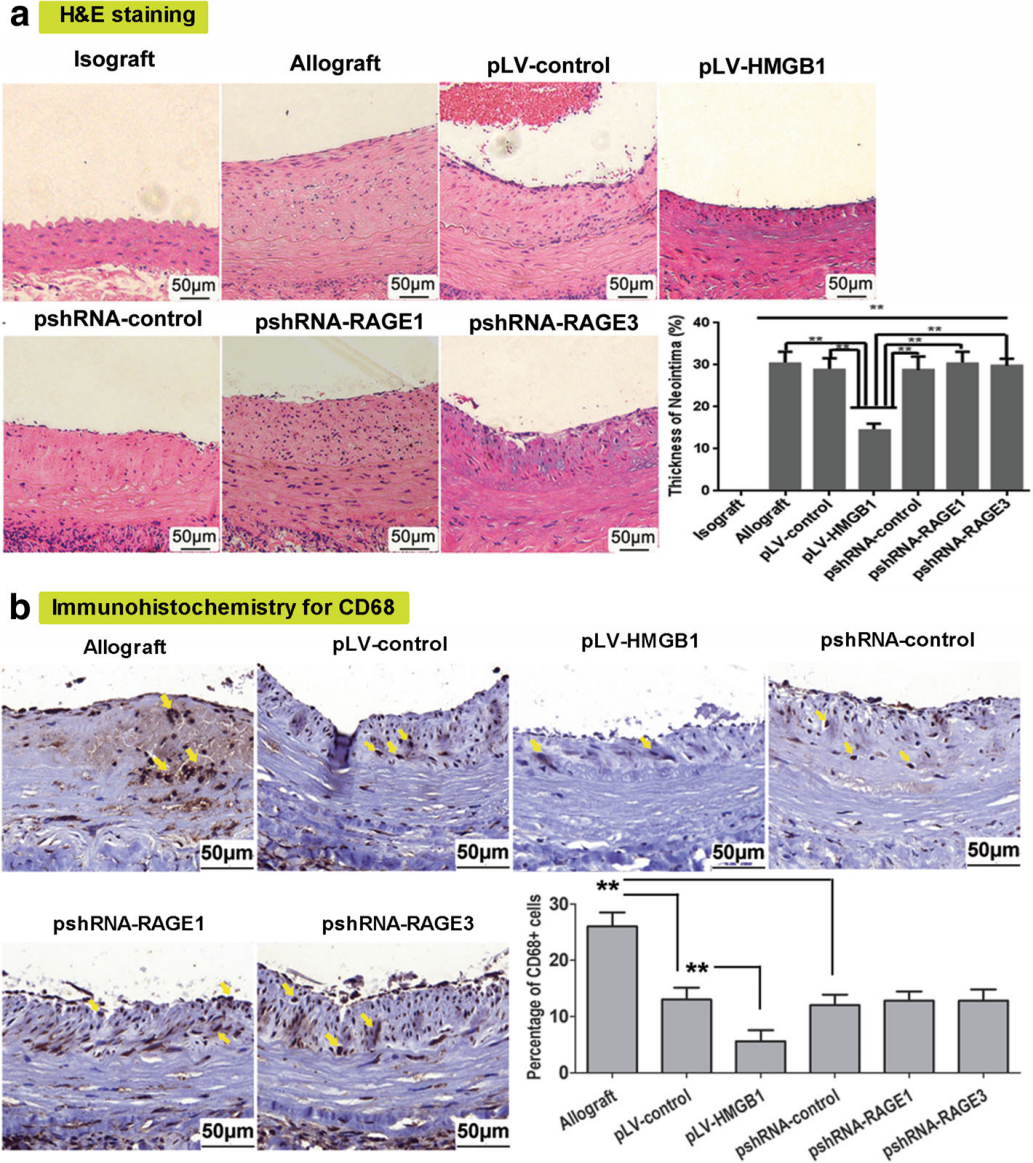
Histological changes in graft vessels 90 days after transplantation. **a** Transplant arteriosclerosis characterized by intimal hyperplasia was observed in all groups except the isograft. Hyperplastic neointima was composed of abundant spindle-shaped cells and extracellular matrix mixed with some degree of inflammation internal to the elastic membrane. Intravenous injection of pLV-HMGB1-transfected MSCs reduced neointimal thickness to the least degree in all allograft groups. However, no favorable effect was elicited by transfection of MSCs with pshRNA-RAGE1 or pshRNA-RAGE3. The neointimal thickness was normalized to the full thickness of vessel walls and expressed as a percentage. Eight rats were examined for each group to calculate the average neointimal thickness. **b** Macrophage infiltration in graft vessels was detected by immunohistochemistry for CD68. Quantitative analysis of CD68^+^ cells in the neointima (marked with yellow arrows) revealed that MSC infusion reduced the fraction of CD68^+^ cells in total neointimal cells. Transfection of pLV-HMGB1 dramatically improved these effects, which were not produced by transfection of either pshRNA-RAGE1 or pshRNA-RAGE3. Five random high-power fields were examined in the sections of graft vessels for each rat, and the neointimal cell number was counted to determine the percentage of CD68^+^ cells in the total cell population. The average percentage was calculated from eight rats for each group. Group comparisons were made using the Mann-Whitney test. **P < 0.05. H&E hematoxylin and eosin, HMGB1 high mobility group box 1, RAGE receptor for advanced glycation end-product

**Fig. 5:**
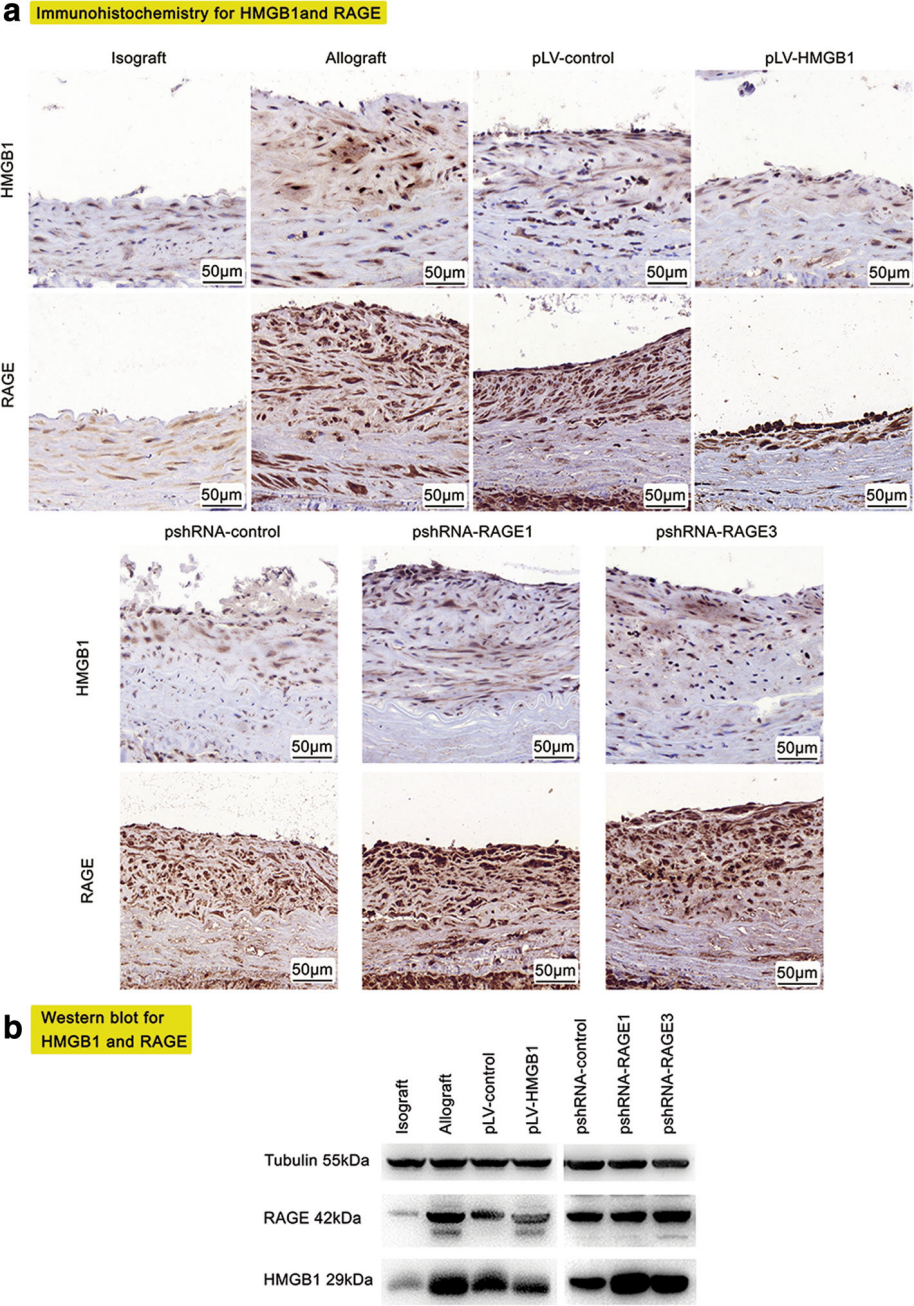
The expression of high mobility group box 1 (HMGB1) and receptor for advanced glycation end-product (RAGE) in graft vessels 90 days after transplantation. **a** Immunohistochemical analysis revealed that both HMGB1 and RAGE were highly expressed in the allograft vessels compared with the isograft. Cytoplasmic expression of HMGB1 was visible in the allografts, suggesting its cytoplasmic translocation and extracellular release. MSC transplantation markedly reduced the expression of HMGB1 and RAGE in allograft vessels. The inhibitory effect was amplified by pLV-HMGB1 transfection, but it was abolished or even reversed by inhibition of RAGE with pshRNA-RAGE1 or pshRNA-RAGE3 transfection. The results were obtained from eight rats for each group. **b** The expression of HMGB1 and RAGE was also analyzed by Western blot. The graft vessels of the same group (eight rats for each group, 50-mg graft vessels for each rat) were mixed together and homogenized to prepare protein samples for Western blot analysis. The results were consistent with those revealed by immunohistochemistry

TA was present in the allografts on the 90th postoperative day. The prominent histological feature was concentric intimal hyperplasia, composed of abundant spindle-shaped cells and associated extracellular matrix mixed with some degree of inflammation internal to the elastic membrane. Transplantation of pLV-HMGB1-transfected MSCs produced significant relief of TA with the least neointimal thickness and macrophage infiltration (CD68^+^ cells in the neointima) among allogeneic transplant groups. However, transfection of cells with pshRNA-RAGE1 and pshRNA-RAGE3 prior to transplantation had little effect (Fig. 4). The sections of graft vessel were immunoassayed for HMGB1 and RAGE. This demonstrated a great number of cells with cytoplasmic expression of HMGB1 and RAGE accumulated within the hyperplasic neointima, which was inhibited by MSC transplantation. Western blot analysis also demonstrated that both HMGB1 and RAGE were highly expressed in the allograft group which was downregulated by MSC transplantation. pLV-HMGB1 transfection improved the ability of MSCs to inhibit HMGB1 and RAGE expression, while the cells transfected with either pshRNA-RAGE1 or pshRNA-RAGE3 exhibited the opposite effects (Fig. 5).

### pLV-HMGB1 transfection promoted MSC migration and endothelial differentiation in vivo and improved endothelial barrier integrity

**Fig. 6:**
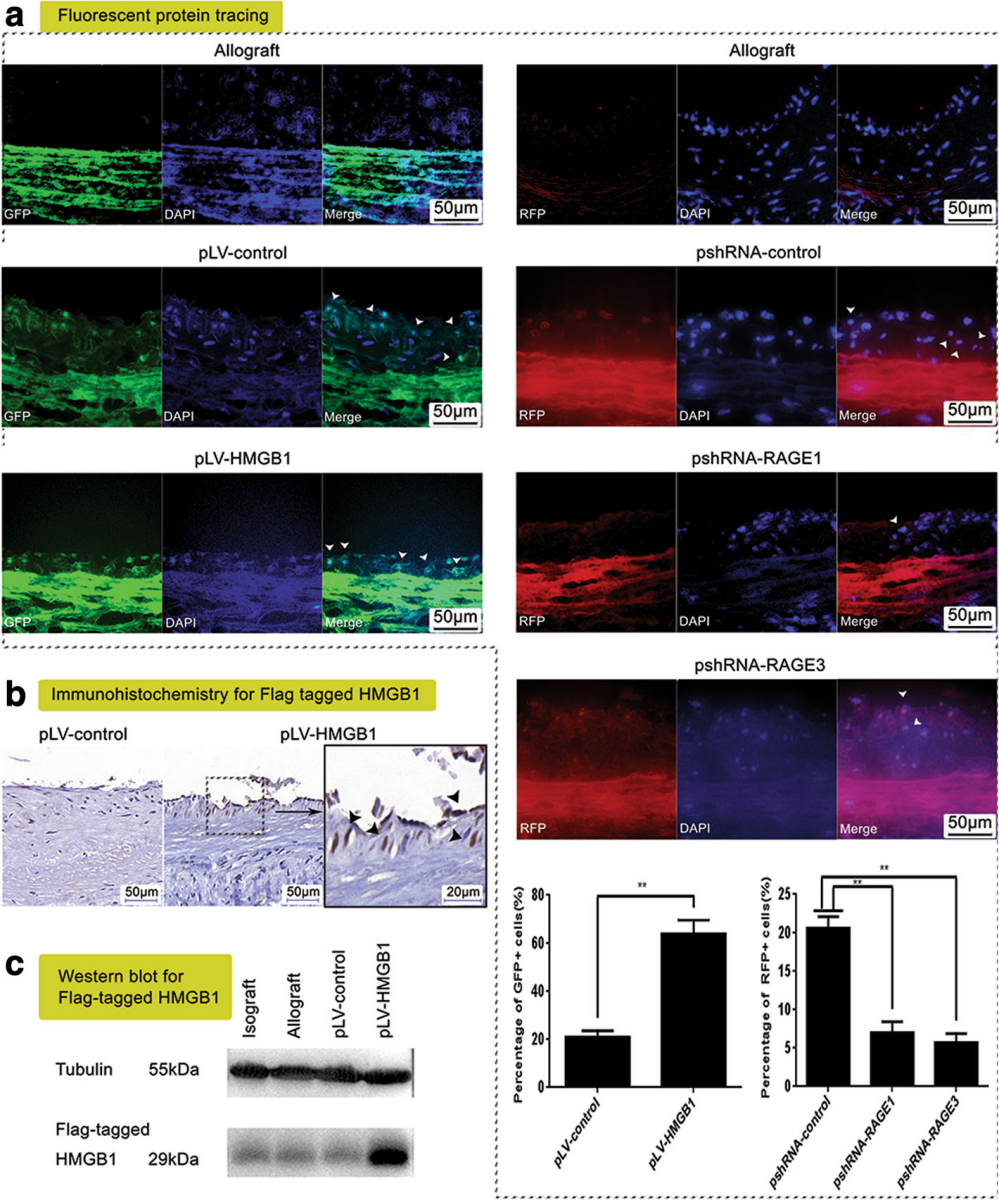
Cell migration to the graft vessels traced by fluorescent protein and Flag tagging. **a** The fluorescent protein-labeled cells (marked with white arrows) were visible in the hyperplastic neointima after MSC transplantation. Five random high-power fields were examined in the sections of graft vessels for each rat, and the neointimal cell number was counted to determine the percentage of fluorescent protein-labeled cells in the total cell population. The average percentage was calculated from eight rats for each group. The percentage of neointimal green fluorescent protein (GFP)^+^ cells was higher in the pLV-HMGB1 group than the pLV-control group. However, the percentage of cherry red fluorescent protein (RFP)^+^ cells was significantly lower in the pshRNA-RAGE1 and pshRNA-RAGE3 groups than in the pshRNA-control group. Group comparisons were made using the Mann-Whitney test. **P < 0.05. **b,c** Cell migration to the neointima was confirmed by immunohistochemistry and Western blot with antibody to Flag-tag in the pLV-HMGB1 group, since Flag-tagged HMGB1 was expressed in the cells transfected with pLV-HMGB1 (marked with black arrows). The view of the graft section represents the results from eight rats (**b**). Homogenate of graft vessels from the same group (eight rats for each group, 50-mg graft vessels for each rat) was prepared for Western blot analysis (**c**). DAPI 4′,6-diamidino-2-phenylindole, HMGB1 high mobility group box 1, RAGE receptor for advanced glycation end-product

**Fig. 7:**
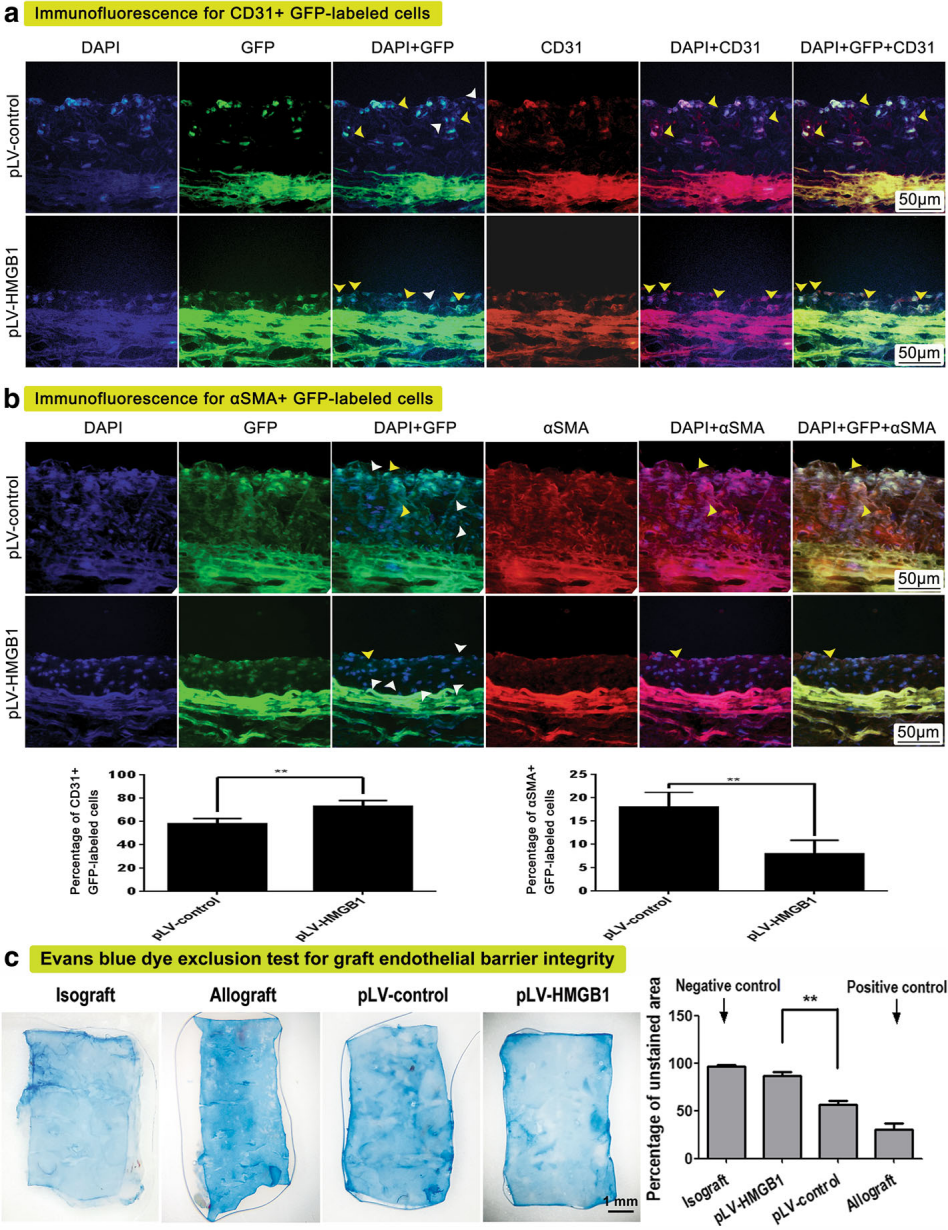
Endothelial differentiation of MSCs in vivo and its association with graft endothelial integrity. **a** Endothelial differentiation of MSCs homed to neointima (green fluorescent protein (GFP)-labeled cells) was assessed by immunofluorescence for CD31. The percentage of neointimal GFP^+^ cells expressing CD31 (marked with yellow arrows) was significantly higher in the pLV-HMGB1 group than in the pLV-control group. **b** α-Smooth muscle actin (αSMA) was selected as the cell maker for MSCs differentiated to SMCs. The fraction of αSMA in neointimal GFP-labeled cells was significantly lower in the pLV-HMGB1 group than in the pLV-control group (marked with yellow arrows). Thus, pLV-HMGB1-transfected MSCs preferably differentiated to the endothelial lineage in vivo. Five random high-power fields were examined in the sections of graft vessels for each rat, and the neointimal cell number was counted to determine the percentage of CD31-positive cells and αSMA-positive cells in the total GFP-labeled cell population. The average percentage was calculated from eight rats for each group. **c** Aortic endothelial barrier integrity was assessed by Evans blue staining in the graft vessels. The area of impaired endothelium was stained with dark blue. Isograft and allograft groups served as negative and positive staining control, respectively. The unstained area was normalized to the total intraluminal surface area of the graft aorta and expressed as a percentage. Compared with the pLV-control group, the percentage of unstained area was significantly increased in the pLV-HMGB1 group. This suggested that infusion of pLV-HMGB1-transfected MSCs had more protective effects on the graft endothelial barrier, which was associated with preferential endothelial differentiation of the cells in vivo. Eight rats were examined for each group. Group comparisons were made using the Mann-Whitney test. **P < 0.05. DAPI 4′,6-diamidino-2-phenylindole, HMGB1 high mobility group box 1, RAGE receptor for advanced glycation end-product

The transplanted cells labeled with fluorescent protein were clearly visible in the allograft neointima. The homing of pLV-HMGB1-transfected cells was verified by immunoassay with antibody to Flag-tag since Flag-tagged HMGB1 was expressed in those cells. The population of fluorescence-labeled cells was counted to calculate the percentage cell composition of graft neointima. Consequently, the fraction of GFP^+^ cells in the pLV-HMGB1 group was greatly increased as compared with the pLV-control group. The percentage of RFP^+^ cells was significantly lower in the pshRNA-RAGE1 and pshRNA-RAGE3 groups than in the pshRNA-control group (Fig. 6). Therefore, MSC recruitment was promoted by pLV-HMGB1 transfection and abolished by RAGE knockdown. The differentiation of GFP^+^ MSCs in the neointima was investigated by immunofluorescence with antibodies to CD31 and αSMA. As a result, the percentage of neointimal GFP^+^ cells expressing CD31 was increased in the pLV-HMGB1 group compared with the pLV-control group, but the αSMA^+^ cells accounted for a relatively smaller proportion of GFP^+^ cells in the pLV-HMGB1 group (Fig. 7). Thus, HMGB1 promoted MSC migration to the allograft neointima and differentiation to the endothelial lineage. This was closely associated with an improved endothelial barrier of graft vessels. The Evans blue staining test revealed that the fraction of unstained area in the endothelium was greatly increased in the pLV-HMGB1 group when compared with the pLV-control group. This suggested that infusion of pLV-HMGB1-transfected cells had more favorable effects on the endothelial barrier.

## Discussion

TA is a common manifestation of chronically rejected grafts which appears with diffuse narrowing and occlusion of the graft vessels caused by concentric neointimal formation. Although it is regarded as a particular type of arteriosclerosis with significant differences to native atherosclerosis, many similarities exist regarding histological changes as well as signaling pathways that are involved, including the HMGB1/RAGE axis [20, 21, 23, 31, 32]. HMGB1 was upregulated in arteriosclerotic plaques and allografts to attract immune cells and stimulate innate and adaptive immunity [15, 23]. Ligation of RAGE by HMGB1 stimulated more production of HMGB1 and further activation of the HMGB1/RAGE axis, forming a vicious cycle to amplify the immune response [33]. In the present study, both HMGB1 and RAGE were highly expressed in allograft vessels, especially in hyperplastic neointima where cytoplasmic translocation of HMGB1 was discovered, suggesting its extracellular release and signaling via RAGE. Despite its proinflammatory nature, some studies have revealed beneficial applications of HMGB1 in injury states. One such application was overexpression of HMGB1 or administration of exogenous HMGB1 to promote tissue remodeling and repair. Extracellular HMGB1 is a potent chemoattractant to recruit regenerative cells to damaged tissue. For instance, more endothelial progenitor cells were recruited and engrafted into ischemic myocardium with transgenic overexpression of HMGB1 than the mock control [34]. The reactive astrocytes in the peri-infarct cortex upregulated HMGB1 which stimulated the migration of endogenous endothelial progenitor cells to promote neurovascular remodeling during stroke recovery [35]. The chemotaxis was at least in part mediated by its principal receptor, RAGE [34, 35]. The present study revealed that MSC migration was enhanced by both exogenous HMGB1 treatment and pLV-HMGB1 transfection. However, the impact of HMGB1 on MSC proliferation is still controversial. An early study showed that HMGB1 inhibited MSC proliferation [36] while the opposite effect was evoked by stimulating MSCs with HMGB1 released from necrotic tumor cells [37]. The present study found that cell growth was inhibited by exogenous HMGB1 treatment as well as pLV-HMGB1 transfection. The previous disagreement was presumably attributable to the variation in experimental conditions. Nevertheless, we demonstrated for the first time that HMGB1 displayed different interactions with VEGF and PDGF, eliciting opposite effects on differentiation of MSCs to ECs and SMCs. HMGB1 inhibited PDGF-induced differentiation of MSCs to SMCs, but it stimulated endothelial differentiation induced by VEGF. Intriguingly, HMGB1 treatment alone failed to induce phenotypic changes (Additional file 4: Figure S3), and there was barely a trace of VEGF or PDGF-BB in the culture supernatant. Similarly, HMGB1 promoted osteogenic differentiation of MSCs, but lost its function in the medium that was devoid of osteoinductive supplements [36]. Thus, HMGB1 regulated MSC differentiation by interacting with other growth factors, and more specifically in our study it promoted VEGF-induced endothelial differentiation but inhibited the differentiation to the SMC phenotype induced by PDGF.

The present study further demonstrates that the regulation of HMGB1 on proliferation, migration, and differentiation of MSCs is through a RAGE-dependent pathway. Firstly, although HMGB1 impeded the growth of MSCs in a dose-dependent manner, the effect was greatly abolished by shRNA-mediated RAGE knockdown. Secondly, RAGE knockdown impaired the stimulatory effect of HMGB1 on endothelial differentiation of MSCs and abolished its inhibition of differentiation to SMCs. Thirdly, RAGE knockdown inhibited MSC migration and impaired the colonization of MSCs in neointima although extracellular HMGB1 as a chemotactic factor was highly expressed in allograft vessels. However, this is contrary to some previous studies that revealed that HMGB1 induced cell migration by forming a complex with CXCL12 and signaling via CXCR4, independent of RAGE pathway [38–40]. Indeed, these are not mutually exclusive; extracellular HMGB1 binds to RAGE to stimulate HMGB1 production, further activating RAGE and more HMGB1 production via a positive feedback loop [33] thus favoring HMGB1-CXCL12/CXCR4-mediated cell migration. Moreover, cell migration to HMGB1 requires nuclear factor κB (NF-κB) activation depending on the inhibitor of NF-κB kinase subunit beta. It is necessary for both CXCR4 and RAGE to maintain their sufficient expression [41], suggesting an intrinsic link between RAGE and CXCR4.

The ability of HMGB1 to stimulate MSCs was translated to a successful application in cell therapy for TA. MSC transplantation holds great promise for the treatment of arteriosclerosis by regulating the immune response as well as generating tissue repair [10, 11]. However, a series of recent studies has shown that vascular resident stem cells acquiring MSC-like phenotype migrated to the endothelium in response to vascular injury and differentiated to SMCs. The stem cells and their differentiated SMCs constituted a major cellular component of the hyperplastic neointima [13, 14, 42–46]. Although stem cells from the vascular walls and other sources might not definitively display a similar differentiation potential, it was suggested that transplantation of unselected or naive MSCs impairs the anti-atherogenic effect or even has an adverse effect. The present study demonstrates that HMGB1-modified MSCs possess a better anti-TA property. Neointimal thickness and macrophage infiltration were dramatically reduced along with a decline in vascular HMGB1 and RAGE expression after the recipients were transplanted with pLV-HMGB1-transfected MSCs. Possible mechanisms are as follows. Firstly, cell migration to allograft vessels was a prerequisite for vascular repair. HMGB1 promoted the migratory capacity of MSCs and stimulated cell recruitment to allograft vessels. The chemotaxis required activation of the HMGB1/RAGE axis since RAGE knockdown impaired MSC migration and produced little therapeutic effect. Secondly, HMGB1 preferably induced the differentiation of MSCs to ECs rather than to SMCs, although both PDGF and VEGF were highly expressed in allograft vessels (Additional file 5: Figure S4). On the one hand, it promoted endothelial regeneration following chronic rejection, which was evidenced by the Evans blue staining test showing that infusion of HMGB1-overexpressing MSCs improved graft endothelial integrity. Theoretically, ECs derived from infused MSCs were isogeneic to the host so as to induce immune tolerance by regenerating endothelium to replace original intima that was allogeneic and rejected by the host immunity. On the other hand, it prevented intimal hyperplasia by inhibiting the recruited MSCs to differentiate to SMCs. If the majority of MSCs differentiated to SMCs, neointimal formation would have been inevitably accelerated by an increased accumulation of SMCs and excessive deposits of extracellular matrix secreted by SMCs in the neointima. Despite its regulation on cell differentiation, HMGB1 slightly inhibited the viability of MSCs. This could threaten cell survival in vivo, but was presumably favorable for improving the outcome of MSC transplantation as the recruited cells were not allowed to proliferate excessively to accelerate intimal hyperplasia. Finally, the immunoregulatory ability of MSCs should not be neglected in cell therapy for TA. It was reported that MSC transplantation promoted the production of regulatory T cells and anti-inflammatory cytokines and inhibited activation of alloreactive immune cells [2–7]. Our study revealed that infusion of HMGB1-overexpressing MSCs attenuated macrophage infiltration in graft neointima. Thus, it will be necessary to investigate the immunological changes after transplantation of HMGB1-modified MSCs in follow-up research.

## Conclusions

The present study demonstrated that the HMGB1/RAGE axis is instrumental in regulating proliferation, migration, and endothelial differentiation of MSCs. HMGB1-modified MSCs were less proliferative than naive MSCs but acquired a better capacity for migration and endothelial differentiation, favoring their application in MSC transplantation for TA.

## Additional files

Additional file 1: Supplemental materials and methods. Construction of lentiviral vectors. Quantitative real-time reverse transcription polymerase chain reaction (qRT-PCR). (DOCX 22 kb)

Additional file 2: **Figure S1.** Modulation of HMGB1 and RAGE expression in MSCs by lentivirus transfection. (TIFF 5432 kb)

Additional file 3: **Figure S2.** Determination of MSC markers by qPCR after viral transfection. (TIFF 21197 kb)

Additional file 4: **Figure S3.** The effect of HMGB1 treatment alone on MSC differentiation. (TIFF 1698 kb)

Additional file 5: **Figure S4.** The expression of VEGF and PDGF-BB in isograft and allograft vessels. (TIFF 2035 kb)

## Abbreviations

DAPI: 4′,6-Diamidino-2-phenylindole
DMEM: Dulbecco’s modified Eagle’s medium
EC: Endothelial cell
FBS: Fetal bovine serum
FITC: Fluorescein isothiocyanate
GFP: Green fluorescent protein
HMGB1: High mobility group box 1
HRP: Horseradish peroxidase
MSC: Mesenchymal stem cell
NF-κB: Nuclear factor κB
OD: Optical density
PDGF: Platelet-derived growth factor
PE: Phycoerythrin
RAGE: Receptor for advanced glycation end-product
RFP: Cherry red fluorescent protein
shRNA: Short hairpin RNA
SMA: Smooth muscle antigen
SMC: Smooth muscle cell
TA: Transplant arteriosclerosis
VEGF: Vascular endothelial growth factor
