# Supplementary material for: The differentiation of mesenchymal stem cells to vascular cells regulated by the HMGB1/RAGE axis: its application in cell therapy for transplant arteriosclerosis
Source: Stem Cell Res Ther. 2018 Apr 3;9:85. doi: 10.1186/s13287-018-0827-z (PMC5883535; doi:10.1186/s13287-018-0827-z)
Supplement: Supplementary file 1 — Supplemental materials and methods. Construction of lentiviral vectors. Quantitative real-time reverse transcription polymerase chain reaction (qRT-PCR). (DOCX 22 kb) [file 13287_2018_827_MOESM1_ESM.docx]

**Supplemental Materials and Methods**

Construction of lentivirus vectors

The lentiviral vectors constructed by Genechem (Shanghai, China) were listed as follows: a pair of vectors which included pLV-HMGB1 to induce HMGB1 overexpression and pLV-control as the negative control, a set of four vectors which consisted of shRNA-RAGE1, shRNA-RAGE2 and shRNA-RAGE3 for RAGE knockdown and pshRNA-control as the negative control. Virus production started by constructing transfer plasmids. The full cDNA sequence of rat HMGB1 was amplified by RT-PCR, and then cloned to a Ubiquitin-MSC-EGFP-3FLAG-IRES-Puromycin GV270 transfer plasmid. The plasmid without the cloning of HMGB1 cDNA served as the negative control. Three oligonucleotides encoding RAGE-targeted short hairpin RNAs (shRNAs) plus a scrambled sequence with no homology of rat genes were synthesized and inserted into 6-MCS-Ubiquitin-Cherry-IRES-Puromycin GV298 transfer plasmid. The target sequences of shRNAs were summarized as follows: 5’-CGTGCAGAGCTGAATCAGT-3’, 5’-GGACTGAAGCTTGGAAGGT-3’ and 5’-GCCGGAAATTGTGAATCCT-3’. The recombinant lentivirus was generated by co-transfecting 293T producer cells with the transfer plasmid and packaging plasmids. Virus particles harvested 72 hours after transfection was purified by passage through a filter of 0.45-μm pore size and then concentrated by centrifugation 4000 × g in Centricon Plus-20 centrifugal filter units (Millipore, Shanghai, China) for 15 min at 4°C. The concentrated virus was serially diluted by culture medium to infect 293T cells for 4 days. The constructed vectors contained fluorescent protein reporter genes. The labeled cells were counted to calculate the transduction unit titer.

Quantitative real time reverse transcription-PCR (qRT-PCR)

Quantitative real time reverse transcription-PCR (qRT-PCR) was performed by using an Eppendorf Mastercycler® (Eppendorf, Hamburg, Germany) to analyze expression of MSC markers including CD29 and CD90. β-actin was used for the internal control. Primers were synthesized by Sangon biotech (Shanghai, China) with sequences shown as follows: CD29 forward sequence: 5’-AATGGAGTGAATGGGACAGG-3’, reverse sequence: 5’-TCTGTGAAGCCCAGAGGTTT-3’. CD90 forward sequence: 5’-AGCTCTTTGATCTGCCGTGT-3’, reverse sequence: 5’-CTGCAGGCAATCCAATTTTT-3’, β-actin forward sequence: 5'-AAGATCCTGACCGAGCGTGG-3', reverse sequence: 5'-GCTAGGAGCCAGGGCAGTA-3'.

**Supplemental Figure Legends**

Figure S1. Modulation of HMGB1 and RAGE expression in MSCs by lentivirus transfection. (A) Over 90% of cells stably expressed fluorescence protein reporter gene (GFP or RFP) after viral transfection. Immunofluorescence assay revealed that the cells transfected with pLV-HMGB1 had higher expression of HMGB1 and RAGE in comparison with pLV-control. RAGE expression was inhibited by cell transfection with pshRNA-RAGE1, pshRNA-RAGE2 and pshRNA-RAGE3. (B) Western blot showed that both HMGB1 and RAGE were highly expressed in pLV-HMGB1-transfected cells. Flag-tagged HMGB1 was also detected in these cells. The expression of RAGE was downregulated in the cells transfected with pshRNA-RAGE1, pshRNA-RAGE2 and pshRNA-RAGE3, while HMGB1 expression had no significant changes.

Figure S3. The effect of HMGB1 treatment alone on MSC differentiation. Naïve MSCs were treated only with 100ng/ml HMGB1 for 14 consecutive days before they were analyzed by flow cytometry. Neither CD31 nor αSMA was detectable on the cell surface.

Figure S4. The expression of VEGF and PDGF-BB in isograft and allograft vessels. Protein was extracted from homogenate of graft vessels by centrifugation. The levels of VEGF and PDGF-BB were detected by using ELISA kits (R&D systems) and normalized to the average levels of isografts. Both growth factors were increased in allografts, approximately 1.5-fold relative to isografts on 90th postoperative day. The results were obtained from three repeated tests for each group. Group comparison was made by using Student t-test. Significant difference was defined as P<0.05 (depicted by asterisks).

Figure S2. Determination of MSC markers by qPCR after viral transfection. CD29 and CD90 as excellent MSC markers were detected by qPCR. Their expression was normalized to that of untreated MSCs and analyzed before and after viral transfection. Consequently, no significant changes were observed after viral transfection. The results were obtained from three repeated tests. Group comparison was made by using Mann-Whitney test. Significant difference was defined as P<0.05.
