# Supplementary figures and images for: The differentiation of mesenchymal stem cells to vascular cells regulated by the HMGB1/RAGE axis: its application in cell therapy for transplant arteriosclerosis
Source: Stem Cell Res Ther. 2018 Apr 3;9:85. doi: 10.1186/s13287-018-0827-z (PMC5883535; doi:10.1186/s13287-018-0827-z)

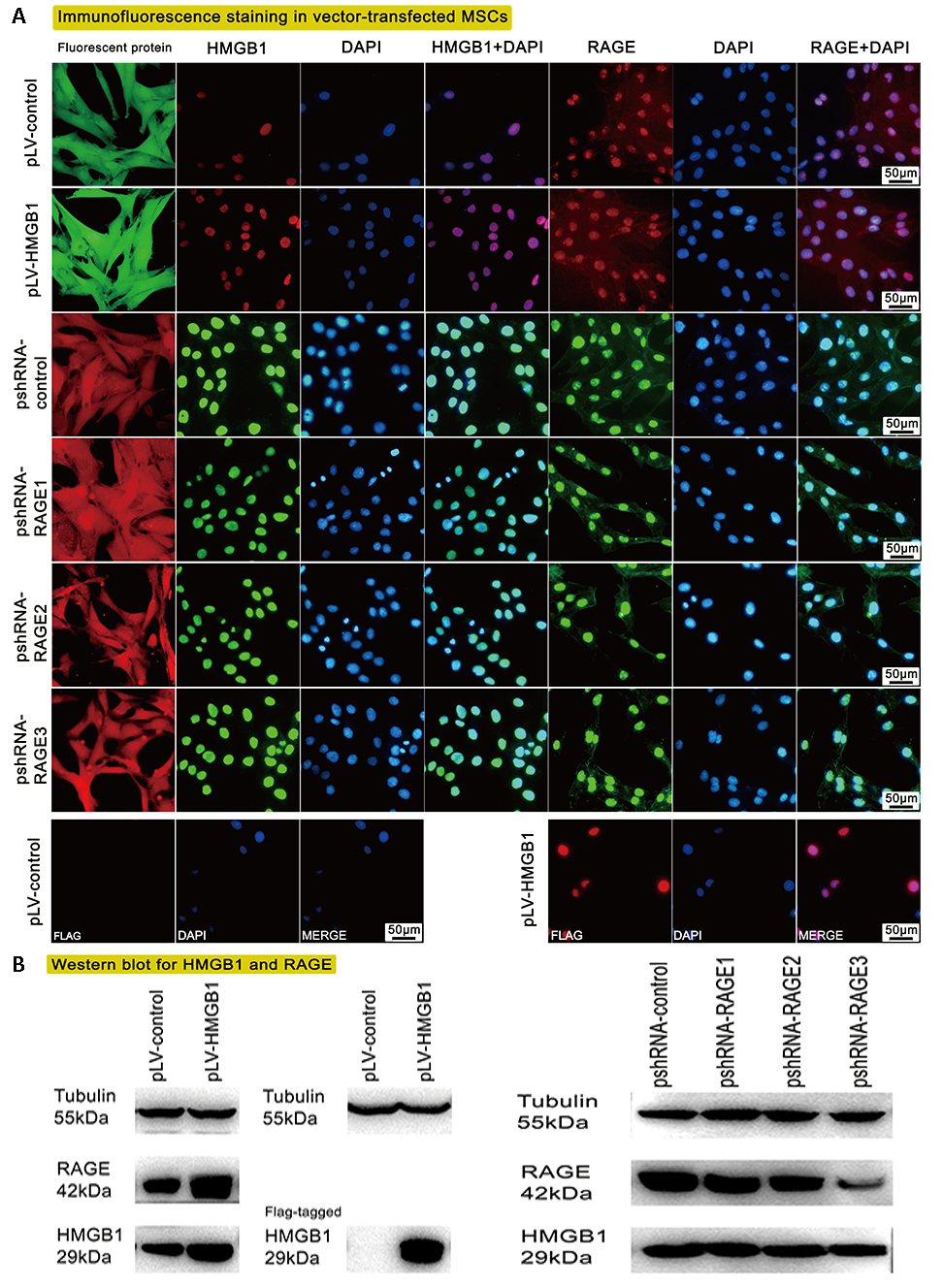

Supplement: Supplementary file 2 — Figure S1. Modulation of HMGB1 and RAGE expression in MSCs by lentivirus transfection. (TIFF 5432 kb) [file 13287_2018_827_MOESM2_ESM.tif]

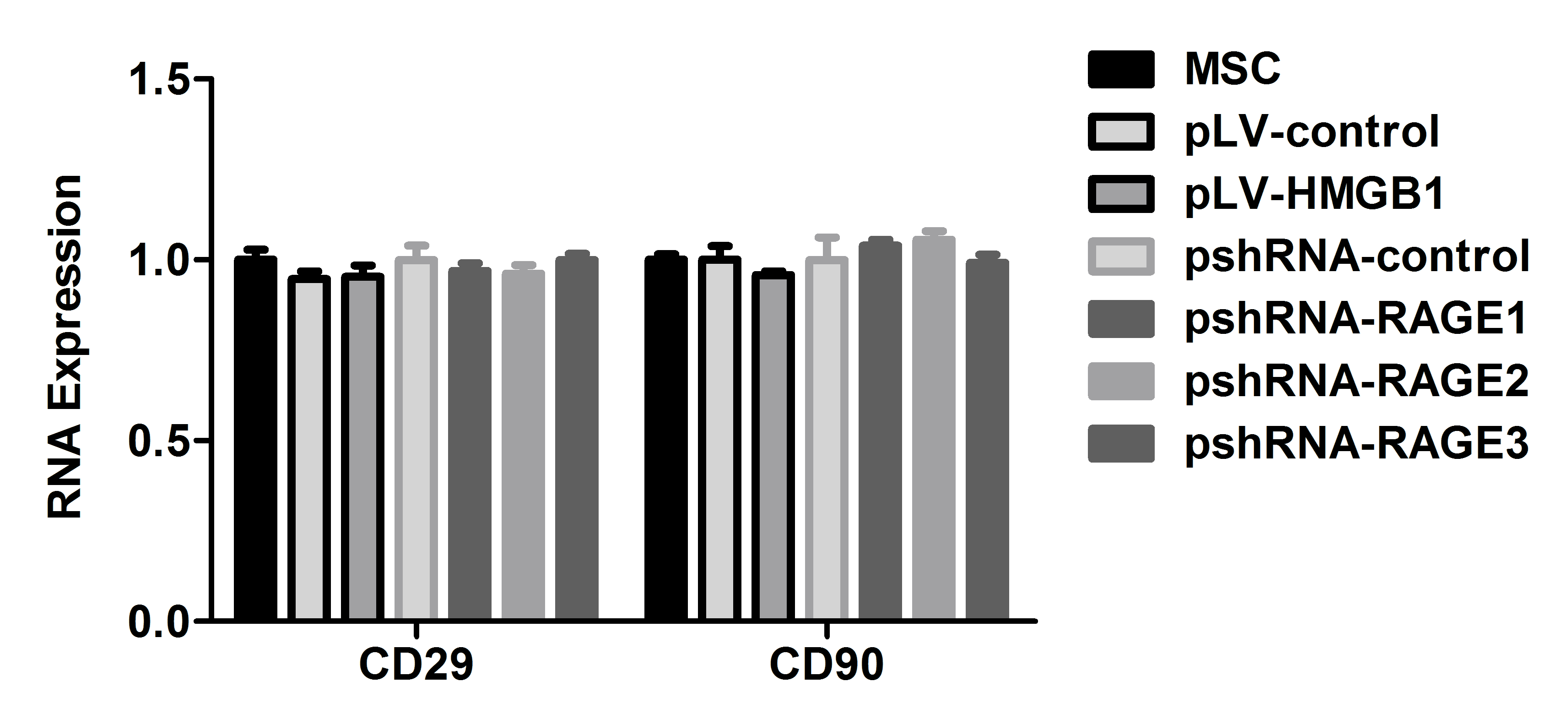

Supplement: Supplementary file 3 — Figure S2. Determination of MSC markers by qPCR after viral transfection. (TIFF 21197 kb) [file 13287_2018_827_MOESM3_ESM.tif]

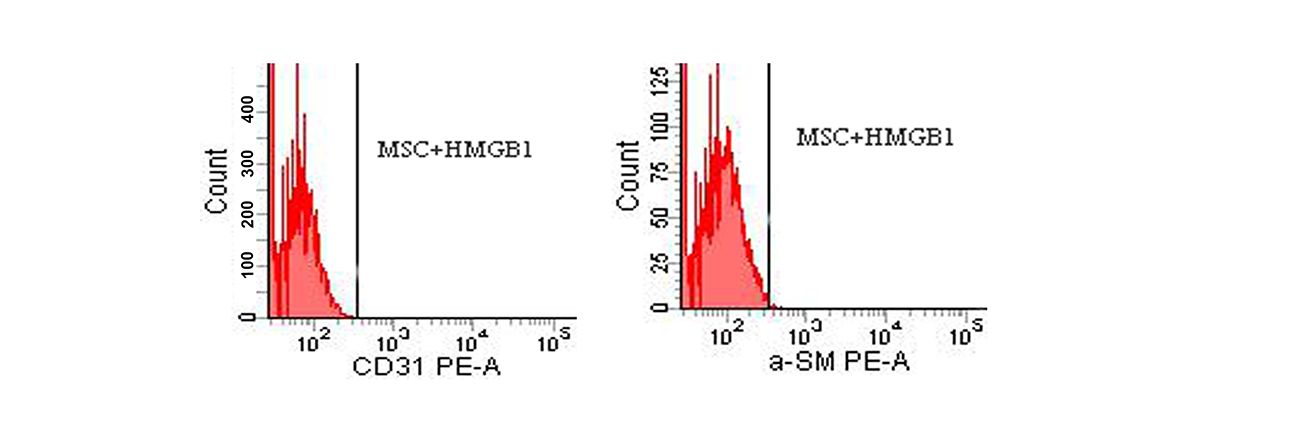

Supplement: Supplementary file 4 — Figure S3. The effect of HMGB1 treatment alone on MSC differentiation. (TIFF 1698 kb) [file 13287_2018_827_MOESM4_ESM.tif]

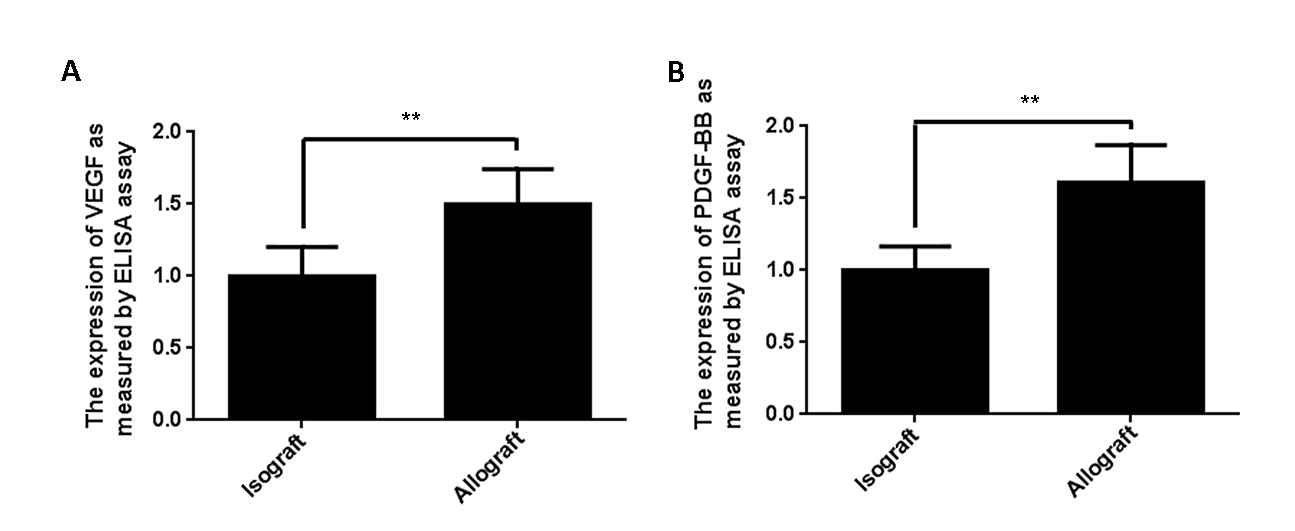

Supplement: Supplementary file 5 — Figure S4. The expression of VEGF and PDGF-BB in isograft and allograft vessels. (TIFF 2035 kb) [file 13287_2018_827_MOESM5_ESM.tif]
